# Olmutinib Reverses Thioacetamide-Induced Cell Cycle Gene Alterations in Mice Liver and Kidney Tissues, While Wheat Germ Treatment Exhibits Limited Efficacy at Gene Level

**DOI:** 10.3390/medicina60040639

**Published:** 2024-04-16

**Authors:** Seema Zargar, Tanveer A. Wani, Salman Alamery, Fatimah Yaseen

**Affiliations:** 1 Department of Biochemistry, College of Science, King Saud University, P.O. Box 22452, Riyadh 11451, Saudi Arabia; salamery@ksu.edu.sa (S.A.); 438204071@student.ksu.edu.sa (F.Y.); 2 Department of Pharmaceutical Chemistry, College of Pharmacy, King Saud University, P.O. Box 2457, Riyadh 11451, Saudi Arabia; twani@ksu.edu.sa

**Keywords:** thioacetamide, liver injury, kidney injury, olmutinib, wheat germ oil, gene expression

## Abstract

*Background and Objectives*: TAA is potent hepatic/renal toxicant. Conversely, WGO is a potent dietary supplement with impressive antioxidant properties. Olmutinib is an apoptotic chemotherapy drug that does not harm the liver or kidney. This study investigated the impact of olmutinib and wheat germ oil (WGO) on Thioacetamide (TAA)-induced gene alterations in mice liver and kidney tissues. *Materials and Methods*: Adult male C57BL/6 mice were exposed to 0.3% TAA in drinking water for 14 days, followed by the oral administration of olmutinib (30 mg/kg) and WGO (1400 mg/kg) for 5 consecutive days. Treatment groups included the following: groups I (control), II (TAA-exposed), III (TAA + olmutinib), IV (TAA + WGO), and V (TAA + olmutinib + WGO). *Results*: The findings revealed that TAA exposure increased MKi67 and CDKN3 gene expression in liver and kidney tissues. Olmutinib treatment effectively reversed these TAA-induced effects, significantly restoring MKi67 and CDKN3 gene expression. WGO also reversed MKi67 effects in the liver but exhibited limited efficacy in reversing CDKN3 gene alterations induced by TAA exposures in both the liver and kidney. TAA exposure showed the tissue-specific expression of TP53, with decreased expression in the liver and increased expression in the kidney. Olmutinib effectively reversed these tissue-specific alterations in TP53 expression. While WGO treatment alone could not reverse the gene alterations induced by TAA exposure, the co-administration of olmutinib and WGO exhibited a remarkable potentiation of therapeutic effects in both the liver and kidney. The gene interaction analysis revealed 77.4% of physical interactions and co-localization between MKi67, CDKN3, and TP53 expressions. Protein–protein interaction networks also demonstrated physical interactions between MKi67, TP53, and CDKN3, forming complexes or signaling cascades. *Conclusions*: It was predicted that the increased expression of the MKi67 gene by TAA leads to the increase in TP53, which negatively regulates the cell cycle via increased CDKN3 expression in kidneys and the restoration of TP53 levels in the liver. These findings contribute to our understanding of the effects of olmutinib and WGO on TAA-induced gene expression changes and highlight their contrasting effects based on cell cycle alterations.

## 1. Introduction

Thioacetamide (TAA) is a well-known hepatotoxin and class B carcinogen, extensively studied for its toxic effects [1,2,3,4]. The impact of TAA exposure on other organs has already been evaluated; however, the effects at the molecular level remain unexplored [5,6,7]. Understanding the molecular mechanisms involved in tissue injury induced by TAA and identifying potential therapeutic strategies are vital for the development of effective treatments. Previous studies have indicated that a single exposure to TAA can temporarily suppress RNA production, followed by a rebound increase in the synthesis of nucleic acids. Even after a single exposure, the noticeable enlargement of the nucleus and increased RNA content can be observed by the third day. Chronic TAA exposure leads to significant elevation in RNA production, accompanied by the enlargement of the nucleus and nucleolus, as well as the accumulation of 45S RNA in these nuclear compartments. These findings suggest potential disruptions in the transport of RNA from the nucleus, increased synthesis, and/or the prolonged retention of RNA within the nucleus [8,9,10].

TP53 is a well-known tumor suppressor protein involved in various cellular processes, including DNA repair, cell cycle regulation, and apoptosis. It has been observed that TP53 can be activated in response to agents that cause damage to DNA. This activation can lead to the suspension of the cell cycle or the initiation of apoptosis, aiming to prevent the spread of damaged cells [11]. The emergence of oxidative stress can induce the upregulation of PTEN expression, inhibit the PI3K/AKT pathway, and eventually lead to cell apoptosis [12]. Therefore, examining the expression and activation status of TP53 in TAA-treated tissues may provide insights into its potential role in mediating the tissue injury response. MKi67 is a protein marker commonly used to assess cell proliferation. It is highly expressed in actively dividing cells and is associated with the cell cycle progression through various stages [13]. Considering the observation of increased RNA synthesis and potential alterations in nucleic acid metabolism in TAA-exposed tissues, it would be relevant to investigate the expression of MKi67. Assessing MKi67 expression could help determine the extent of cellular proliferation or aberrant cell cycle progression in response to TAA exposure. CDKN3 (Cyclin-Dependent Kinase Inhibitor 3) is a protein involved in regulating the cell cycle by inhibiting cyclin-dependent kinases. Alterations in CDKN3 expression or activity can impact cell cycle progression and may be indicative of dysregulated cell division. Considering the potential disruption in RNA transport and increased RNA synthesis observed in TAA-exposed tissues, it would be valuable to explore the expression levels and potential perturbations in CDKN3. Assessing CDKN3 expression could provide insights into its involvement in cell cycle regulation and potential dysregulation in response to TAA exposure.

Olmutinib, a targeted therapy commonly used in the treatment of lung cancer, has shown promising results in reversing gene alterations associated with cancer progression [14,15]. However, its potential efficacy in counteracting TAA-induced gene alterations in non-cancerous tissues remains largely unexplored. On the other hand, wheat germ, a nutrient-rich component derived from wheat grains, has been recognized for its potential health benefits [16,17]. In our previous in silico analysis, WGO showed anti-inflammatory activities via FABP4 interacting physically with target genes (77.84%) and co-expressing with 8.01% genes [18]. Nevertheless, its ability to mitigate TAA-induced gene alterations has not been well investigated at cellular levels. 

Understanding how olmutinib and wheat germ oil affect gene expression and related markers in TAA-induced liver and kidney injury can provide valuable insights into their potential therapeutic benefits with respect to the cell cycle. Elucidating the underlying molecular mechanisms and identifying effective interventions for TAA-induced tissue injury holds significant clinical relevance, as it could pave the way for novel treatment strategies. By investigating the impact of olmutinib and wheat germ on gene alterations and related markers, we aim to shed light on their potential as therapeutic interventions for non-cancerous or cancerous tissue injury induced by TAA. Such findings may contribute to the development of targeted therapies and nutritional interventions that can mitigate the detrimental effects of TAA exposure on liver and kidney tissues. MKi67 is expressed during all active phases of the cell cycle (G1, S, G2, and mitosis) but is absent in resting cells (G0). The increased expression of MKi67 is often observed in proliferating cells and is associated with a higher growth rate of cancer cells and poorer prognosis [19]. The TP53 gene encodes the TP53 protein, a well-studied tumor suppressor. TP53 plays a crucial role in regulating cell cycle progression, DNA repair, and apoptosis. Mutations or the dysregulation of TP53 can lead to uncontrolled cell growth and contribute to the development of various cancers [20]. CDKN3 encodes the cyclin-dependent kinase inhibitor 3, which acts as a negative regulator of the cell cycle by inhibiting cyclin-dependent kinases. The dysregulation of CDKN3 can lead to uncontrolled proliferation and contribute to cancer development [21]. Overall, this study seeks to expand our understanding of the molecular responses to TAA exposure and explore the potential of olmutinib and wheat germ oil as therapeutic options for TAA-induced liver and kidney injury. The results obtained from this research may have implications for clinical practice and the development of personalized approaches to mitigate the adverse effects of TAA exposure.

Our previous study investigated the protective effect of WGO and olmutinib on the TAA-induced toxicity mouse model by various biochemical and inflammatory markers, including histological studies. The previous findings demonstrated that the toxic effects of TAA were enhanced by olmutinib, while WGO exhibited a protective effect. Further, it was also concluded that WGO is a non-toxic dietary supplement that can reduce liver and kidney damage caused by TAA and olmutinib in mice [1]. In this study, we aimed to investigate the effects of olmutinib and wheat germ oil, alone and in combination, on the gene alterations induced by TAA exposure in mouse liver and kidney tissues. We focused on assessing the expression levels of MKi67, TP53, and CDKN3 genes, which are associated with cell proliferation, tumor suppression, and cell cycle regulation, respectively [22,23,24]. 

## 2. Materials and Methods

### 2.1. Animal Model and Experimental Design

A total of thirty mice, aged between 4 and 6 weeks and weighing approximately 19 g (with a variation of ±1 g), were housed at the animal care unit located at the College of Medicine, KSU. The mice were randomly divided into different groups: control group, TAA-exposed group, TAA-exposed group treated with olmutinib, and TAA-exposed group treated with wheat germ oil. The dosage, administration method, and duration of TAA exposure followed the established protocol are outlined in our previous study [1]. To ensure compliance with ethical standards, the mice were humanely euthanized using carbon dioxide asphyxiation, in accordance with the guidelines provided by NC3Rs. The research received approval from the Institutional Animal Ethics Committee, with the assigned ethical reference number KSU-SE-21-08 (approval date 6 December 2020). After euthanasia, the liver and kidneys were carefully removed, rinsed with saline solution, weighed, and sectioned into smaller portions. These tissue samples were then immersed in RNAlater solution (Qiagen, Hilden, Germany) to prevent RNA degradation and were subsequently stored at a temperature of −80 °C until RNA extraction was performed.

### 2.2. RNA Extraction

Approximately 30 mg of tissue was placed into a sterile microcentrifuge tube, and 350 μL of RNA Lysis Buffer (Promega, Madison, WI, USA), already containing beta-mercaptoethanol, was added. The mixture was then homogenized, and the lysate was centrifuged for 3 min at 14,000 rpm. The resulting supernatant was carefully transferred to a new microcentrifuge tube. To the cleared lysate, 350 μL of 70% ethanol was added and mixed thoroughly by pipetting. This solution was then loaded onto an RNeasy spin column placed in a 2 mL collection tube and was centrifuged at 12,000× *g* for 15 s. The flow-through was discarded, and 700 μL of RNA Wash Solution was applied to the spin column, followed by centrifugation at 10,000 rpm for 15 s. After that, 500 μL of Buffer RPE was added to the column, which was then centrifuged for 2 min at the same speed to wash the RNA. The flow-through was discarded, and the RNeasy spin column was transferred to a fresh 2 mL collection tube and centrifuged at 14,000 rpm for 1 min to dry the column membrane. For the final step of RNA elution, 30 μL of RNase-free water was directly added to the spin column membrane, and the column was placed in a new 1.5 mL collection tube. The tube was centrifuged for 1 min at 10,000 rpm to elute the RNA [25]. The purity and concentration of the extracted RNA were assessed using a NanoDrop Lite Spectrophotometer (Thermo Scientific, Waltham, MA, USA). The absorbance ratio (260/280 nm = 2) was used as an indicator to assess the purity of the RNA.

### 2.3. cDNA Synthesis

The total RNA was reverse transcribed into DNA under standard conditions, using SuperScript^®^ III First-Strand Synthesis System for RT-PCR Kit (Thermo Fisher Scientific, Waltman, MA, USA), according to the manufacturer’s protocol instructions and appropriate dilutions [26].

### 2.4. Gene Expression Analysis by Real-Time PCR

The gene expression levels of MKI67, TP53, and CDKN3 genes were measured using qRT-PCR by quantifying the transcribed mRNA (cDNA). For qPCR, the SYBR^®^ Green Kit (QIAGEN, Venlo, The Netherlands) was used. The relative gene expression values of mRNA transcripts were calculated after normalizing to the values of the GAPDH housekeeping gene and relative control samples. The data were analyzed using the comparative threshold cycle method (2^−ΔΔCT^) [27]. The sequences of the primers of a target gene and GAPDH (reference/internal control gene) are listed in Table 1.

### 2.5. Co-Expression Network Analysis

Co-expression network analysis and protein–protein interaction (PPI) assessment are powerful methods used to uncover gene modules and identify groups of genes with similar biological functions or regulatory relationships. In this study, we employed GeneMANIA (https://genemania.org/, accessed on 13 December 2023) for co-expression network analysis and the STRING database (https://stringdb.org, accessed on 13 December 2023) to analyze PPIs, along with confidence scores of 1, indicating the reliability of the interactions. The combination of these techniques enabled us to gain insights into the functional relationships and potential regulatory mechanisms of genes within specific biological contexts [28].

### 2.6. Statistical Analysis

Data obtained from gene expression analysis were analyzed using appropriate statistical methods. Statistical significance between groups was determined using *t*-tests, ANOVA, or non-parametric tests, depending on the data distribution and experimental design. *p*-values less than a predetermined significance level (e.g., *p* < 0.05) were considered statistically significant.

## 3. Results

In our research, we conducted a comprehensive analysis of the effects of olmutinib and wheat germ oil (WGO) on gene expression markers TP53, MKi67, and CDKN in the liver and kidney tissues exposed to thioacetamide (TAA). These gene markers play crucial roles in tumor suppression, cell proliferation, and cell cycle regulation, making them important targets for assessing the impact of therapeutic interventions.

**Figure 1:**
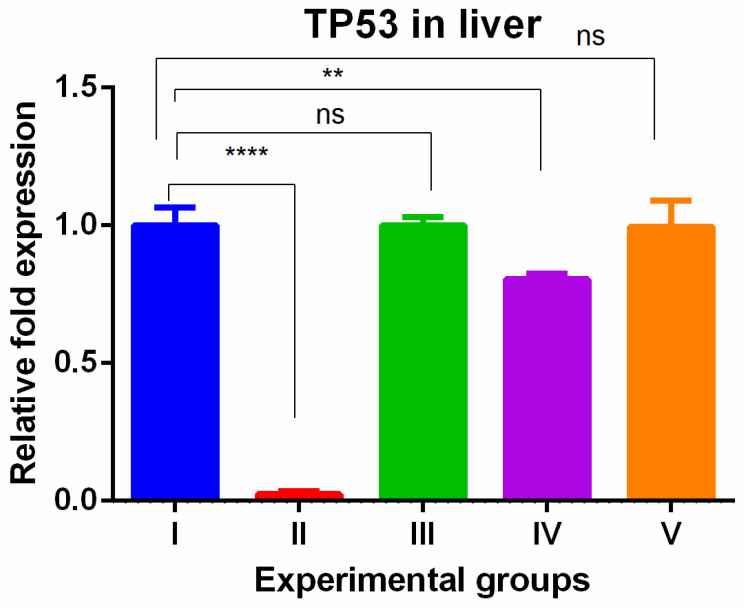
Relative gene expression levels of cell cycle and apoptotic marker TP53 in the liver. The expression levels were assessed using qPCR in mice induced with TAA and treated with olmutinib and WGO alone and in combination. Values are represented as mean ± SD of six mice in each group; ** is *p* < 0.05; ns is non-significant and **** is *p* < 0.0005.

The level of TP53 gene expression showed tissue specific expression levels in the liver and kidney tissues. In the liver, the TAA-induced group showed drastically lower gene expression compared to the control group. The olmutinib treatment reversed the drastic decrease in tumor suppressor gene in the group treated by olmutinib alone or in combination with WGO. In contrast, there was no protective effect shown in the group (group IV) treated with WGO alone, but in the combination group (V), the therapeutic effect of olmutinib was potentiated by WGO (Figure 1). 

**Figure 2:**
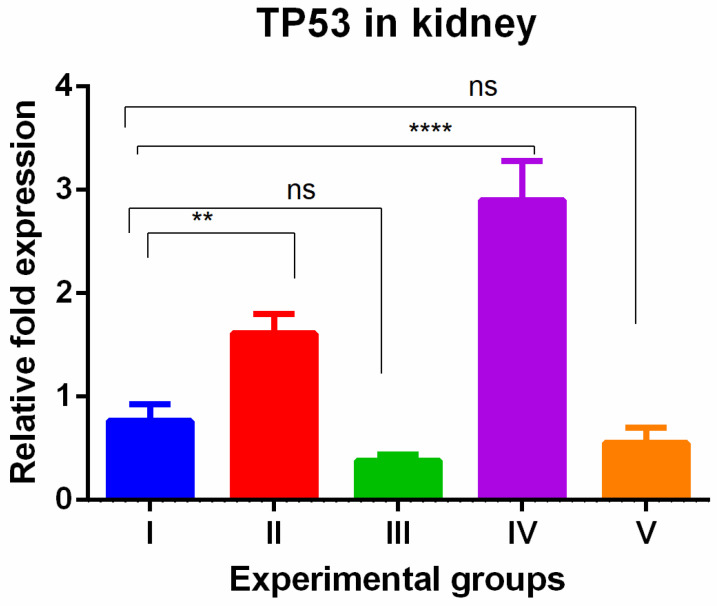
Relative gene expression levels of cell cycle and apoptotic marker TP53 in the kidney. The expression levels were assessed using qPCR in mice induced with TAA and treated with olmutinib and WGO alone and in combination. Values are represented as mean ± SD of six mice in each group; ** is *p* < 0.05; ns is a non-significant change; and **** is *p* < 0.0005.

In the kidney, a different pattern of tissue-specific expression was observed with the highest TP53 gene expression in TAA-induced group and WGO-treated TAA groups that was reversed completely by olmutinib-treated groups, and to our surprise, the olmutinib effect was further potentiated in combination group. This phenomenon could be because of hormesis. Hormesis is a biological response characterized by a beneficial or stimulatory effect at low levels of exposure or stress, followed by a detrimental effect at higher levels (Figure 2). 

**Figure 3:**
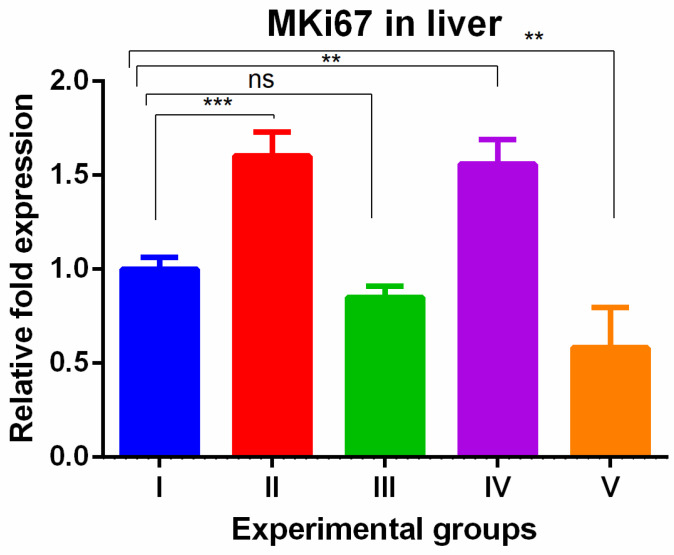
Relative gene expression levels of the cell cycle proliferation marker MKi67 in the liver. The expression levels were assessed using qPCR in mice induced with TAA and treated with olmutinib and WGO alone and in combination. Values are represented as mean ± SD of six mice in each group; ** is *p* < 0.05; ns is a non-significant change; and *** is *p* < 0.005.

The MKi67 gene also showed higher gene levels in TAA and WGO groups (groups II and IV) and the increased levels were reversed in the TAA group treated with olmutinib alone (groups III) and the protective effect was potentiated in combination group of liver tissue (group V). The potentiation was significantly low when compared to the control (Figure 3). 

**Figure 4:**
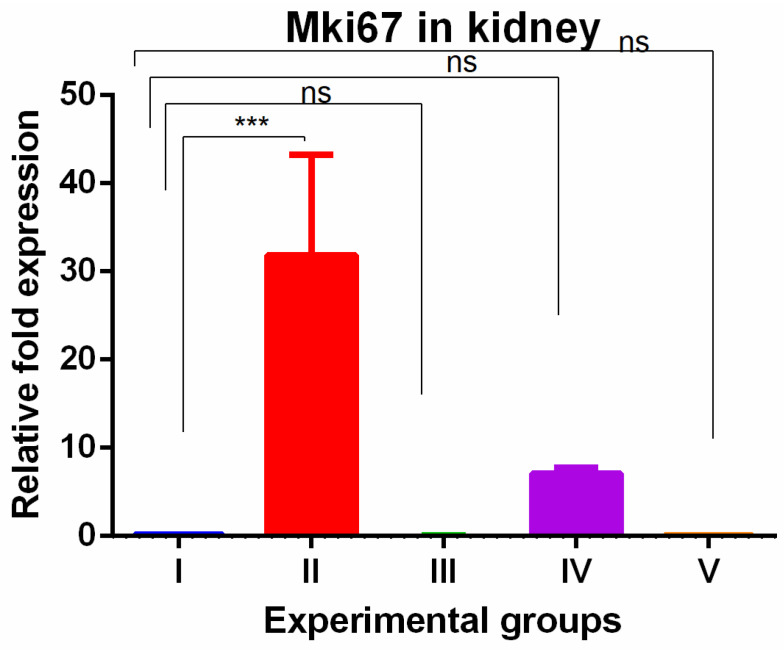
Relative gene expression levels of cell cycle proliferation marker MKi67 in kidneys. The expression levels were assessed using qPCR in mice induced with TAA and treated with olmutinib and WGO alone and in combination. Values are represented as mean ± SD of six mice in each group; ns is a non-significant change and *** is *p* < 0.005.

The kidney tissue showed significant increased MKi67 expression with TAA exposures which were completely reversed by WGO as well as olmutinib with the expression significantly lower when compared to TAA-induced group (Figure 4). 

**Figure 5:**
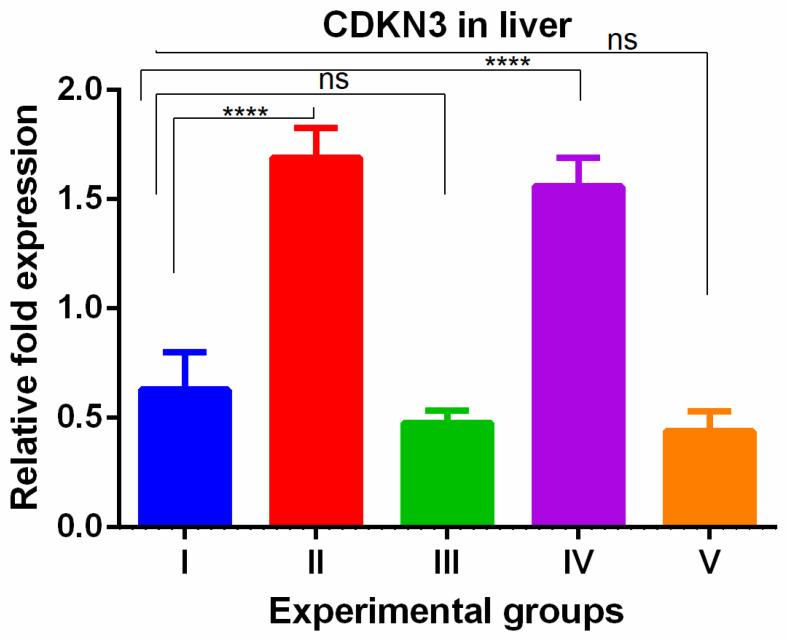
Relative gene expression levels of cell cycle regulation marker CDKN3 in the liver. The expression levels were assessed using qPCR in mice induced with TAA and treated with olmutinib and WGO alone and in combination. Values are represented as mean ± SD of six mice in each group; ns is a non-significant change and **** is *p* < 0.0005.

WGO was having protective effect in kidney with respect to cell proliferation. The CDKN3 gene showed a significant increase in the livers of thioacetamide-treated mice which was reversed in all olmutinib groups (Figure 5). 

**Figure 6:**
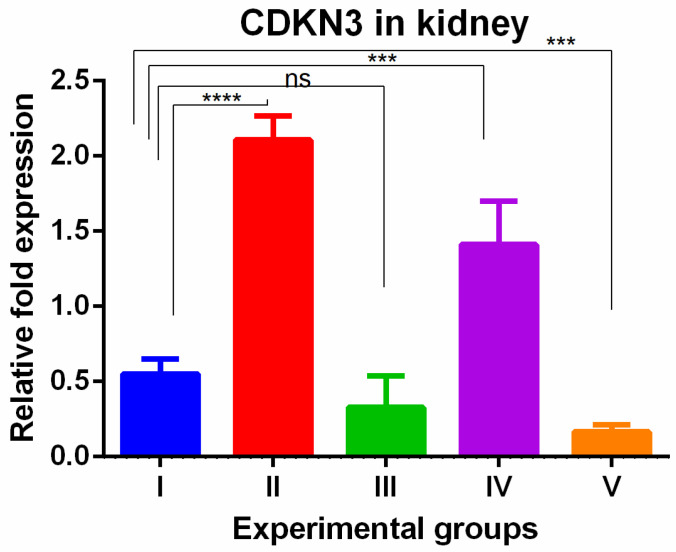
Relative gene expression levels of cell cycle regulation marker CDKN3 in the kidney. The expression levels were assessed using qPCR in mice induced with TAA and treated with olmutinib and WGO alone and in combination. Values are represented as mean ± SD of six mice in each group; ns is non-significant change, **** is *p* < 0.0005 and *** is *p* < 0.005.

A significant increase in CDKN3 was shown in the kidneys of TAA-induced groups. WGO administration (group IV) non-significantly decreased these changes partially while the olmutinib group (group III) reversed them completely when compared to control group. The combination group decreased the levels to below normal (group V), which can be attributed to hormesis (Figure 6). 

**Figure 7:**
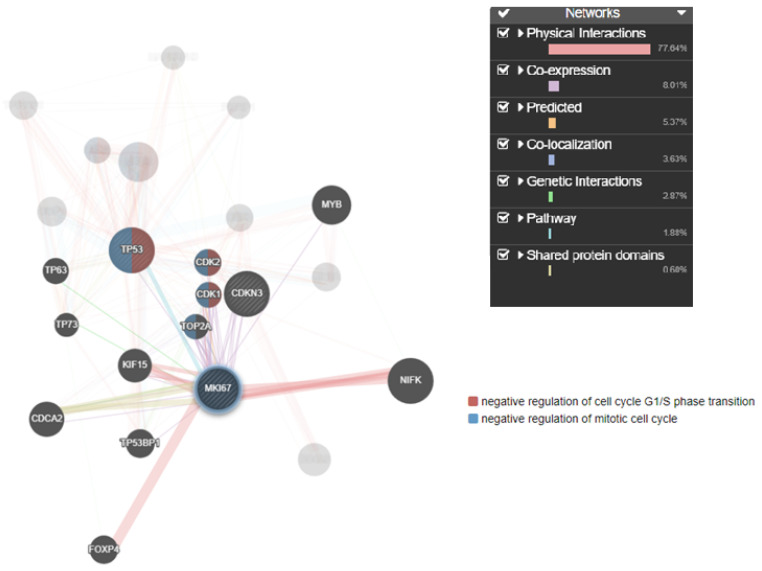
Target genes and their interactions linked to TAA, olmutinib, and WGO action. Genes involved in cell cycle regulation, apoptosis, and proliferation showed direct interaction by the TP53 regulator inducing CDK1, a negative regulator of mitotic cell cycle transition through MKi67. The thickness of the nodes represents the strength of the interaction. The green highlighted genes are directly involved in mitotic cell cycle transition.

Understanding the complex interplay between genes and their products is crucial for unraveling the underlying mechanisms of various biological processes. Co-expression network analysis provided a systematic approach to identify modules or clusters of genes that were co-regulated and hence likely to be involved in similar biological functions or pathways. Additionally, assessing protein–protein interactions elucidated the molecular interactions and functional relationships between TP53, MKi67, and CDKN3 proteins. The gene interaction results showed the co-localization of MKi67, CDKN3, and TP53. CDKN3 is a cell cycle regulator that controls the transition from G2 phase to mitosis (M phase) by dephosphorylating cyclin-dependent kinases (CDKs) and thereby helps regulate the cell cycle progression (Figure 7). 

**Figure 8:**
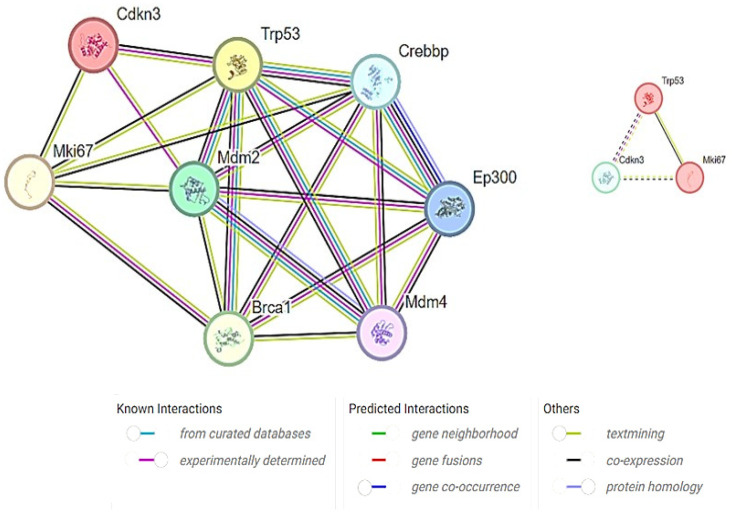
Target proteins and their interactions linked to TAA, olmutinib, and WGO action. Proteins involved in cell cycle regulation, apoptosis, and proliferation showed direct interaction by TP53 protein fusion with the Mki67 protein, inducing CDKN3, a negative regulator of mitotic cell cycle transition. All the three proteins showed coexpression, suggesting their interactions. The color key represents the strengths and types of the interactions.

CDKN3 has been implicated in various cellular processes, including cell proliferation, cell migration, and cancer development. Furthermore, a predefined set of genes exhibited statistically significant protein–protein interactions. Mouse species-specific PPI networks illustrated experimentally determined interactions as well as the co-expression between MKi67, TP53, and CDKN3. All the three proteins directly interacted with each other, forming complexes or signaling cascades (Figure 8). GeneMANIA has depicted the role of TP53 in mitotic cell cycle phase transition and G1/S phase transition. Hence, it was predicted that the increased expression of the MKi67 gene leads to the increase in TP53, which negatively regulates the cell cycle via increased CDKN3 expression.

## 4. Discussion

In the liver tissue, exposure to TAA resulted in a notable decrease in the expression of the TP53 gene compared to the control group. However, when olmutinib was administered, this decrease was reversed, indicating that olmutinib may have a protective effect on TP53 gene expression. This discovery aligns with previous studies that highlight the importance of TP53 in suppressing tumor growth and how its dysfunction contributes to the development of tumors [29]. Interestingly, treatment with WGO alone did not exhibit a protective effect on TP53 gene expression, suggesting that WGO may not directly impact this tumor suppressor pathway. Conversely, olmutinib treatment successfully restored TP53 expression levels in both the olmutinib-only group and the olmutinib combined with WGO group. These results align with a previous meta-analysis that reported a decreased clinical efficacy of EGFR-TKIs in the presence of concurrent TP53 mutations [30]. Our study further supports the notion that olmutinib, a well-known TKI, exerts its effects by increasing TP53 expression. In the kidney tissue, the TAA-induced group and the WGO-treated TAA groups exhibited higher TP53 gene expression, which was completely reversed by olmutinib treatment. This indicates that both TAA and WGO may have influenced TP53 gene expression in the kidneys, and olmutinib effectively counteracted these effects. This finding is consistent with the role of olmutinib in restoring TP53 function and potentially preventing damage in the kidneys. Moreover, our recent research demonstrates the therapeutic synergistic action of poziotinib and olmutinib through the activation of the TP53-dependent apoptotic pathway [28]. Our findings have two important implications. Firstly, they highlight the ability of olmutinib to protect against the decrease in TP53 expression. This suggests that olmutinib may have a protective effect on TP53, which is a crucial gene involved in tumor suppression. Secondly, our results indicate that olmutinib has the potential to enhance the effectiveness of tumor suppression pathways. These insights are significant as they can guide the development of targeted therapies that specifically aim to utilize TP53’s role in the treatment of cancer.

The increased MKi67 gene expression in the TAA group was completely reversed in the kidneys and was partially reversed in the liver by both WGO and olmutinib treatment. This suggests that both interventions effectively suppressed cell proliferation in the kidneys and liver, indicating their potential protective effects. It is worth noting that WGO has been reported to possess antioxidant properties and may contribute to tissue protection, but at gene level, it shows limited efficacy [1]. This observation is significant because it emphasizes that substances with antioxidant properties can still provide protective effects, even if their influence on gene expression may not be as pronounced. In line with these results, Gu et al. created a nomogram based on MKi67 expression to predict how well first-line therapy works in people with non-small cell lung cancer (NSCLC). According to their research, patients with lower levels of MKi67 expression had a much better chance of survival, while patients with higher levels of MKi67 expression had a worse prognosis (OS) [31]. This suggests that MKi67 could be an important biomarker for cancer progression and therapeutic outcomes. Also, new studies have shown that high MKi67 levels may be a sign of aggressive disease progression in people who are being treated with first-line EGFR-TKIs [32]. Recently, another study reported that a high expression of MKi67 may indicate the aggressive progression to the first-line EGFR-TKI. Overall, the results indicate that olmutinib, likely through its EGFR-TKI action, and WGO, through its antioxidant capacity, can modulate cell proliferation as measured by MKi67 expression.

The changes in CDKN3 gene expression seen with different treatments suggest that these interventions may regulate the cell cycle in the liver and kidneys. In the liver, olmutinib reversed the increased CDKN3 expression caused by TAA exposure and WGO treatment. This indicates that olmutinib may protect against CDKN3 overexpression in the liver, affecting cell cycle control. In the kidneys, TAA exposure led to increased CDKN3 expression, which was partially reduced by WGO but completely reversed by olmutinib. This suggests that olmutinib may have a stronger impact on CDKN3 regulation and cell cycle inhibition in the kidneys compared to WGO. These findings are important because CDKN3 plays a role in various cellular processes. CDKN3 plays a crucial role in various cellular processes, such as tumor cell proliferation, DNA replication, cell invasion and migration, and apoptosis. It is often found to be overexpressed in different types of cancers and is associated with poor prognosis [21,33,34]. The correlation between high CDKN3 expression and reduced survival rates emphasizes the importance of this gene as a potential target for cancer therapy. The ability of olmutinib to decrease CDKN3 expression could be a valuable therapeutic mechanism, potentially contributing to the inhibition of tumor growth and progression.

Previous research identified TP53, MKi67, and CDKN3 as central genes in the pathology of hepatocellular carcinoma. Our study’s PPI network and gene interaction analysis also pinpoint these genes as pivotal elements within the kidney [35]. To find out how MKi67, TP53, and CDKN3 are connected, we made mouse-specific PPI networks that included both confirmed interactions and co-expression data. The networks showed direct links between the three proteins, which could mean that they work together to form complexes or take part in signaling pathways. The gene interaction study from experimental data unveiled a notable co-occurrence of MKi67 and TP53 within the cellular environment. MKi67 is closely linked to cell proliferation because it is a known marker of cellular division. TP53 is an important gene that serves as a tumor suppressor and is essential for many cellular functions, including controlling the cell cycle and fixing DNA. The coexistence of these genes suggests a probable interdependence, which may suggest that they operate in concert to regulate biological processes. The combination of GeneMANIA and the Cytoscape plugin has emerged as a powerful approach for studying the intricate network of interactions between genes and proteins and to construct visual models that predict the collective functions of genes that govern various cellular activities [36]. This approach is effective in unraveling the underlying mechanisms and relationships between genes on induction by xenobiotics or environmental toxins. It may provide a deeper understanding of their roles in biological processes.

The study also found a large network of protein–protein interactions involving TP53, MKi67, and CDKN3. CDKN3 is an important cell cycle regulator that affects chemosensitivity by controlling the change from the G2 phase to the M phase [21]. By influencing cyclin-dependent kinases, which are required for appropriate cell cycle progression and must be dephosphorylated by CDKN3, olmutinib attains chemosensitivity on TAA-induced cells. This interaction shows a possible way that olmutinib and WGO might work to control things; it involves proteins TP53 and MKi67 that affect CDKN3. This implies that the precise moment cells enter the mitotic phase is coordinated with cell proliferation regulation by these proteins.

The study shows that olmutinib treatment significantly changes the expression of TP53 and CDKN genes in liver tissue, which shows that it works to stop cells from multiplying and changes the cell cycle. In contrast, renal tissues did not exhibit this therapeutic effect, indicating that the medication elicits a distinct response in other organs. On the other hand, WGO showed that it could slow down changes in the liver and protect the kidneys by keeping MKi67 expression high. It is crucial to acknowledge that the protective effect of WGO was not all-encompassing and did not affect all affected genes, including those in the CDKN family. This suggests that WGO has a selective effect on particular genes and its effectiveness may be limited in specific organs. The changes in gene expression with olmutinib are similar to those with other chemotherapeutic agents [37,38]. Targeted activities have the potential to induce both direct and indirect alterations in patterns of gene expression. As a dietary supplement, WGO, which is well-known for its antioxidant effects, is probably not involved in the direct regulation of gene expression. Its principal role is to mitigate oxidative stress by the elimination of free radicals. Although antioxidants like WGO have the ability to modulate cell signaling pathways, which results in an indirect influence on gene expression, their effects are typically more nuanced in comparison to targeted chemotherapeutic drugs such as olmutinib. This study has substantially improved our understanding of complicated regulatory networks and functional links in co-expressed gene clusters. Further investigation is needed to understand how these genes interact and how the treatments influence these pathways by immunoprecipitation (IP) or co-immunoprecipitation (Co-IP) experiments. This will directly identify physical interactions between MKi67, CDKN3, and TP53 proteins that would provide stronger evidence for the formation of complexes or signaling cascades suggested by this study. Furthermore, potential confounding factors like animal age, dose response testing, environmental stress, and the normalization of gene expression data and tissue processing can be controlled to ensure the confidence of these findings in future studies. This will assist us in understanding how these drugs interact with biological systems and in assessing their efficacy and safety for clinical use.

## 5. Conclusions

Our study shows that olmutinib treatment significantly affects liver and kidney TP53 and CDKN gene expression, confirming its anti-proliferative and cell cycle regulating actions. The administration of WGO showed varying degrees of protection, with kidney tissues exhibiting the complete preservation of MKi67 expression compared to partial protection in the liver. These data imply that while olmutinib has a broad and potent effect on essential genes involved in cell proliferation and cell cycle control, WGO may give a more limited protection, depending on tissue type and individual genes. The interaction analysis revealed that increased MKi67 expression may increase TP53 levels. TP53 could restrict the cell cycle by upregulating CDKN3 expression, which controls cell proliferation and cycle regulation. More research is needed to comprehend olmutinib and WGO’s complex gene expression effects and their potential as TAA-induced liver and kidney injury treatments. GeneMANIA depicted the role of TP53 in mitotic cell cycle phase transition and G1/S phase transition. To further investigate the role of TP53 in cell cycle regulation and its specific effect post-olmutinib treatment, future studies could consider cell cycle analysis, gene expression profiling, and functional assays to elucidate the mechanisms underlying the anti-proliferation effects of olmutinib and the involvement of TP53 in cell cycle regulation.

## Figures and Tables

**Table 1:** The forward and reverse primer sequences of the target genes.

Name of Gene	Primers		Primer Name
MKi67	Forward	AAGAAGAGCCCACAGCACAGAGAA	M_MKi67-E15F
	Reverse	AAGAAGAGCCCACAGCACAGAGAA	M_MKi67-E16R
CDKN3	Forward	TTCTGCCATTCTCACCGTGTCCTT	M_CDKN3-E4F
	Reverse	TGCGATAACAAGCTCCGTCCATCT	M_CDKN3-E6R
TP53	Forward	AACAATGGCCCGAGTCTAATGGGA	M_TP53-E2F
	Reverse	ACAGATGTTGCCTGATGTCTGGGT	M_TP53-E4R
GAPDH	Forward	ACCACAGTCCATGCCATCAC	M_GAPDH-F
	Reverse	ACCACAGTCCATGCCATCAC	M_GAPDH-R

## Data Availability

The data will be available upon reasonable request.
